# Combined targeting of Raf and Mek synergistically inhibits tumorigenesis in triple negative breast cancer model systems

**DOI:** 10.18632/oncotarget.20534

**Published:** 2017-08-24

**Authors:** Teddy S. Nagaria, Changnian Shi, Charles Leduc, Victoria Hoskin, Soma Sikdar, Waheed Sangrar, Peter A. Greer

**Affiliations:** ^1^ Division of Cancer Biology and Genetics, Cancer Research Institute, Queen's University, Kingston, ON, Canada; ^2^ Department of Pathology and Molecular Medicine, Queen's University, Kingston, ON, Canada

**Keywords:** triple negative breast cancer, Raf, Mek, selumitinib, synergy

## Abstract

Aberrant Ras-MAPK signaling from receptor tyrosine kinases (RTKs), including epidermal growth factor receptor (EGFR) and human epidermal growth factor receptor-2 (HER2), is a hallmark of triple negative breast cancer (TNBC); thus providing rationale for targeting the Ras-MAPK pathway. Components of this EGFR/HER2-Ras-Raf-Mek-Erk pathway were co-targeted in the MDA-MB-231 and MDA-MB-468 human TNBC cell lines, and *in vitro* effects on signaling and cytotoxicity, as well as *in vivo* effects on xenograft tumor growth and metastasis were assessed. The dual EGFR/HER2 inhibitor lapatinib (LPN) displayed greater cytotoxic potency and MAPK signaling inhibition than the EGFR inhibitor erlotinib, suggesting both EGFR and HER2 contribute to MAPK signaling in this TNBC model. The Raf inhibitor sorafenib (SFN) or the Mek inhibitor U0126 suppressed MAPK signaling to a greater extent than LPN; which correlated with greater cytotoxic potency of SFN, but not U0126. However, U0126 potentiated the cytotoxic efficacy of LPN and SFN in an additive and synergistic manner, respectively. This in-series Raf-Mek co-targeting synergy was recapitulated in orthotopic mouse xenografts, where SFN and the Mek inhibitor selumitinib (AZD6244) inhibited primary tumor growth and pulmonary metastasis. Raf and Mek co-inhibition exhibits synergy in TNBC models and represent a promising combination therapy for this aggressive breast cancer type.

## INTRODUCTION

The receptor tyrosine kinases (RTKs) EGFR and HER2 have been widely associated with breast cancer (BC) pathogenesis. EGFR is over-expressed and genetically amplified in one-third of metastatic or recurrent BCs, and this has been inversely correlated with relapse-free survival [1–5]. HER2 overexpression is detected in 20% of BC cases and is strongly associated with poor clinical prognosis [6]. HER2 has been established as a relevant prognostic and predictive biomarker for BC, and as a target in both advanced and early stages of BC [6–8].

EGFR and HER2 have been implicated in promoting tumor cell proliferation and survival through the Ras-Raf-Mek-Erk (Ras-MAPK) pathway [9–11]. Delayed tumor onset in a transgenic mouse model of HER2^+^ breast cancer correlated with impaired Ras-mediated Erk activation, which argues that Ras-MAPK signaling from EGFR-HER2 potentiates tumor development and progression [12, 13]. Elevated Ras-MAPK signaling in BC has been associated with advanced clinical stage, including metastatic lymph node infiltration and insensitivity to hormone therapy [14]. Targeted therapies such as the anti-HER2 monoclonal antibody Trastuzumab and the dual anti-EGFR/HER2 tyrosine kinase inhibitor lapatinib (LPN) have been developed to mitigate pathogenic signaling from these RTKs. Trastuzumab has been approved for treatment for HER2+ BC [15–17], and LPN for advanced/metastatic cases [18].

Raf and Mek are also established targets for inhibiting oncogenic signals arising from upstream RTKs or from gain-of function mutations in *RAS* or *RAF* that drive Ras-MAPK signaling [11, 19, 20]. Sorafenib (SFN) (BAY 43-9006; Nexavar), which potently inhibits both the c-Raf (Raf-1) and b-Raf isoforms, was approved by the FDA in 2005 for treatment of advanced renal cell carcinoma [11]. SFN prolonged progression free survival (PFS) of advanced or metastatic HER2 negative BC patients (SOLTI-0701) [21] and advanced HER2 negative patients with disease progression during or after Bevacizumab treatment (NCT00493636 trial) [22]. U0126 and PD98059 were among the first generation of Mek inhibitors developed to inhibit MAPK signaling but were abandoned due to poor pharmacodynamics and metabolic instability [11]. Selumitinib (AZD6244; ARRY-142886), a recent generation non-ATP competitive inhibitor of Mek1/2, displayed preclinical anti-tumorigenic effects in colorectal carcinoma, non-small cell lung cancer (NSCLC) melanoma and BC [11, 23, 24]. AZD6244 improved overall survival (OS) and PFS in advanced *KRAS*-mutant NSCLC patients (NCT00890825), supporting Mek inhibition as a strategy for disrupting Erk signaling, irrespective of upstream stimuli [25].

Triple negative breast cancer (TNBC) is a highly aggressive form of BC, as indicated by poor survival rates relative to other forms of BC [26, 27]. Adjuvant chemotherapy is the current standard treatment for TNBC, but modest response rates underscores a need for more effective treatments. TNBC is characterized by lack of over-expressed estrogen receptor (ER), progesterone receptor (PR), or the HER2 receptor tyrosine kinase [28], so therapeutics targeting these oncogenic drivers are not clinically indicated, as they are for ER^+^ or HER2^+^ BCs. As observed in BC and other carcinomas, elevated levels of activated Erk kinases have also been reported in TNBC [14, 29], suggesting important survival and mitogenic regulatory roles for this pathway. Interestingly, molecular alterations in components of the Ras-MAPK pathway are not as frequently observed in TNBC [30] as they are in other cancer types. However, EGFR is frequently expressed in TNBC. In addition HER2 is expressed in basal-like BCs which can compose 70-90% of clinical TNBCs [31], EGFR activates Ras-MAPK signaling in TNBC [32], and in some TNBC populations this could be effected through HER2-dependent mechanisms. These observations provide rationale for exploring these RTKs and components of the downstream Ras-MAPK signaling pathway as therapeutic targets in TNBC.

Targeted agents developed to inhibit EGFR, HER2, Raf and Mek have elicited significant clinical responses in cancer patients; however relapse and resistance is typically observed [33, 34]. This has prompted the notion of using combinations of targeted agents designed to block both the primary and secondary targets that might mediate resistance [35]. In this study, we explored the molecular, cytotoxic and anti-tumorigenic effects of co-targeting EGFR, HER2, Raf and Mek in the MDA-MB-231 TNBC cell line model. *In vitro* co-targeting of EGFR and HER2 using LPN was more effective than targeting EGFR alone with erlotinib, and additive cytotoxicity was observed when Raf or Mek inhibition was combined with EGFR/HER2 co-targeting. Mek inhibition significantly potentiated SFN-induced cytotoxic efficacy *in vitro*; and combined inhibition of Mek and Raf with AZD6244 and SFN almost completely abolished tumor growth and pulmonary metastasis *in vivo*. Importantly, cell killing efficacy was enhanced at low doses of the latter inhibitors. These data highlight the potential of in-series co-targeting of MAPK signaling pathway components to improve efficacy and minimize adverse toxicity in TNBC treatment.

## RESULTS

### Targeting EGFR in TNBC cells induced poor Ras-MAPK signal inhibition and cytotoxicity

**Figure 1:**
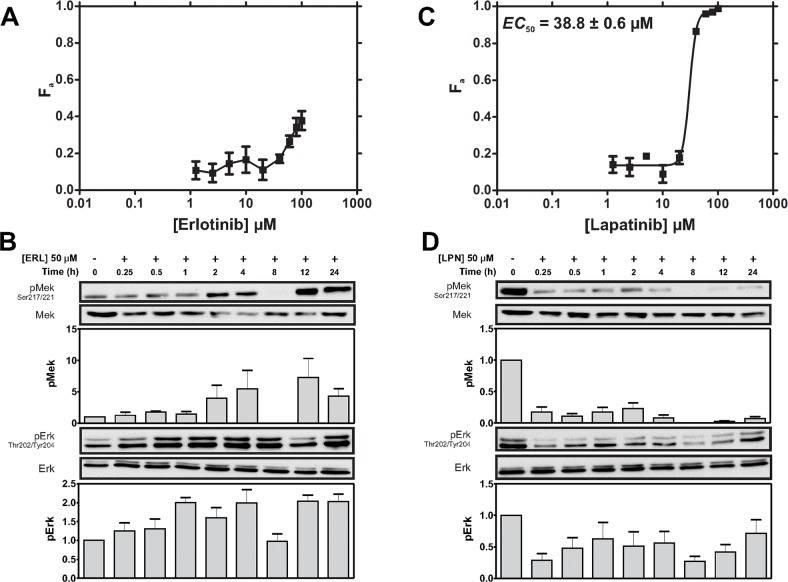
MDA-MB-231 cells display greater cytotoxic sensitivity to LPN compared to ERL **(A)** Cells were treated with ERL (0-100μM) and cell viability was assayed after 72 h. The fraction of cells killed (F_a_ ± SEM) is shown. An *EC_50_* was not determined because of the low F_a_ did not reach saturation in the tested concentration range. The cytotoxicity profile is representative of 3 independent experiments. **(B)** Cells were treated with ERL (50 μM) for 24 h. Mek phosphorylation at Ser217/221 (pMek) and Erk phosphorylation (pErk) at Thr202/Tyr204 were assessed by immunoblotting (IB). Loading was assessed by IB for total Mek and Erk. Representative IBs and density analysis of pMek and pErk IB experiments are shown. pErk and pMek are expressed as a ratio of Mek and Erk intensity values, respectively (mean ± SEM; pMek/Mek, *n* = 2; pErk/Erk, *n* = 4). **(C)** Cells were treated with LPN (0-100 μM) and F_a_ was assayed after 72 h. The LPN *EC*_50_ is was estimated to be 38.8 ± 0.6 μM using non-linear regression analysis. The cytotoxicity profile is representative of 3 independent experiments. **(D)** Cells were treated with LPN (50 μM) for 24 h and pMek and pErk phosphorylation were determined by IB. Representative IBs and density analysis of Mek and Erk experiments are shown. pErk and pMek are expressed as a ratio of Mek and Erk intensity values, respectively (mean ± SEM; pMek/Mek, *n* = 2; pErk/Erk, *n* = 4).

The EGFR inhibitor erlotinib (ERL) was initially used to assess the impact of targeting EGFR on MDA-MB-231 cell viability and downstream Ras-MAPK signaling (as assessed by Mek and Erk phosphorylation). ERL achieved less than 50% kill efficacy (*K_Eff_*; maximal percentage of cells killed) at 100 μM (Figure 1A). Limited *K_Eff_* correlated with the failure of ERL to inhibit downstream Ras-MAPK signaling at concentrations as high 50 μM (Figure 1B); and with an unexpected increase in Ras-MAPK signaling (Figure 1B).

### Co-targeting EGFR and HER2 induced greater MAPK signal inhibition and cytotoxicity than targeting EGFR alone in TNBC cells

MDA-MB-231 cells also express HER2, the preferred heterodimerization partner of EGFR [36]; thus survival signals in MDA-MB-231 cells may be propagated through EGFR-HER2 heterodimers instead of, or in addition to EGFR-EGFR homodimers. This suggested greater *K_Eff_* might be achieved with the dual kinase EGFR/HER2 inhibitor LPN [37]. Indeed, LPN achieved over 95% *K*_Eff_ at 100 μM, with an *EC*_50_ (dose required to achieve half-maximal cell killing) of 38.8 μM (Figure 1C). This enhanced cytotoxicity (relative to ERL) correlated with efficient inhibition of Mek and Erk phosphorylation (Figure 1D). Interestingly, weak Mek and Erk signal recovery was apparent at 1-4 and 24 h indicating that these kinases remain functionally coupled during the tested period of inhibition (Figure 1D). Taken together, these observations suggest co-inhibition of both EGFR and HER2 is required to efficiently induce cytotoxicity in MDA-MB-231 TNBC cells.

### Potent SFN-mediated cytotoxicity correlated with strong MAPK signal inhibition in TNBC cells

**Figure 2:**
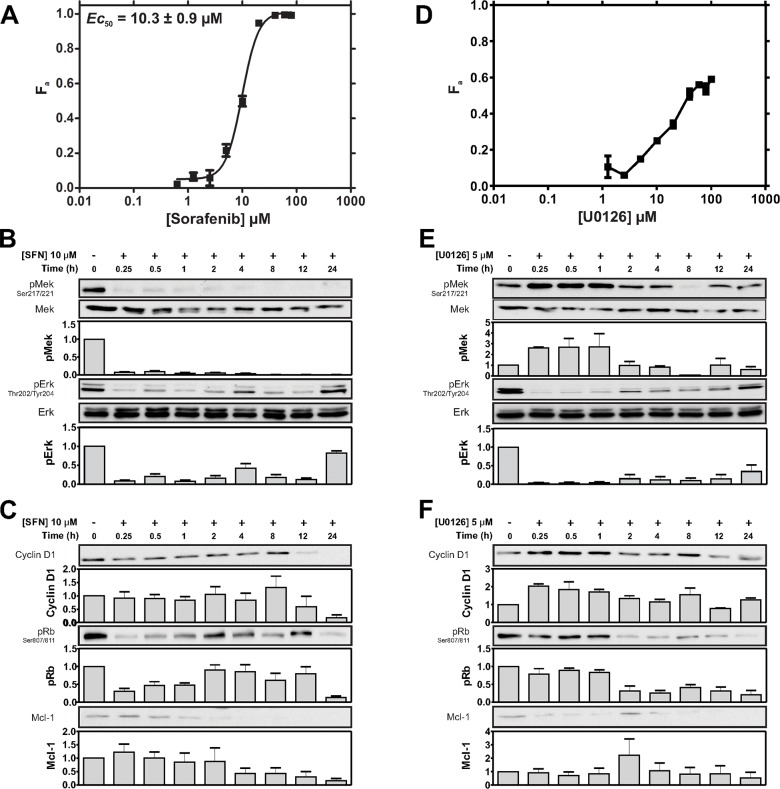
Cytotoxic sensitivity to the Raf inhibitor SFN and the MEK inhibitor U0126 **(A)** Cells were treated with SFN (0-80 μM) and viability was assayed after 72 h. The SFN *EC*_50_ is was estimated to be 10.3 ± 0.9 μM. The cytotoxicity profile is representative of 3 independent experiments. **(B)** Cells were treated with SFN (10 μM) for 24 h and the indicated proteins or phosphoproteins were assessed by IB. Representative IBs and density analysis of pMek and pErk IBs are shown. pErk and pMek are expressed as a ratio of Mek and Erk intensity values, respectively (mean ± SEM; pMek/Mek, *n* = 2; pErk/Erk, *n* = 4). **(C)** Representative IBs of Rb phosphorylation (at Ser807/811), Cyclin D1 and, Mcl-1 in response to 10 μM SFN. Corresponding density analyses of Rb, Cyclin D1 and Mcl-1 IBs expressed as a ratio of RasGAP intensity levels are shown below (mean ± SEM; pRb, *n* = 2; Cyclin D1, *n* = 2 Mcl-1, *n* = 2). **(D)** Cells were treated with U0126 (0-100 μM) and viability was assayed after 72 h. An *EC_50_* was not determined because of the low F_a_. The cytotoxicity profile is representative of 3 independent experiments. **(E)** Cells were treated with U0126 (5 μM) for 24 h and the indicated proteins or phosphoproteins were assessed by IB. Representative IBs and density analysis of pMek and pErk IBs are shown. pErk and pMek are expressed as a ratio of Mek and Erk intensity values, respectively (mean ± SEM; pMek/Mek, *n* = 2; pErk/Erk, *n* = 3). **(F)** Representative IBs showing Rb signaling (as assessed by S807/S811 phosphorylation), as well as Cyclin D1 and, Mcl-1 expression are shown in response to 5 μM U0126. Corresponding density analyses of Rb, Cyclin D1 and Mcl-1 IBs expressed as a ratio of RasGAP intensity levels are shown below (mean ± SEM; pRb/RasGAP, *n* = 2; Cyclin D1/RasGAP *n* = 2; Mcl-1/RasGAP, *n* = 2).

We next explored targeting downstream components of the Ras-MAPK pathway. The multi-kinase inhibitor SFN was used to target Raf. SFN killed 95% of MDA-MB-231 cells at 20 μM, with an *EC*_50_ of 10.3 μM (Figure 2A). This cytotoxic effect is almost 4-fold more potent than LPN (compare to LPN *EC_50_* of 38.8 μM; Figure 1C). In addition, SFN strongly suppressed Mek and Erk phosphorylation at 10 μM (Figure 2B); a 5-fold lower concentration than that used for LPN (50 μM), (Figure 1D). As with LPN, SFN inhibited Erk phosphorylation between 0.25-12 hours, with evidence of periodic signal recovery at 4 and 24 hours (Figure 2B). These data indicate that targeting Ras-MAPK signaling is slightly more effective with SFN than with LPN; and secondly, this correlates with improved cytotoxic potency relative to upstream EGFR and HER2 co-targeting.

### SFN-mediated cytotoxicity correlated with suppressive effects on Rb, CyclinD1 and Mcl1 signaling

MAPK signaling has been implicated in regulating mitogenesis through promoting cell-cycle entry through transcriptional induction of Cyclin D1 [38]. Steady-state Cyclin D1 expression was largely unaffected during the first 8 hours of SFN treatment, but rapidly declined thereafter; suggesting that SFN has a delayed anti-mitogenic effect in these cells (Figure 2C). Effects on cell-entry may be mediated by Cyclin D1-mediated activation of the retinoblastoma (Rb)-E2F pathway, which is implicated in regulating both mitogenesis and survival [39]. Interestingly, SFN transiently suppressed Rb phosphorylation between 0.25-1 hours; however pRb signal recovered between 2-12 hours, before rapidly declining again at 24 hours (Figure 2C). Lastly, SFN also suppressed expression of Mcl-1, a pro-survival member of the Bcl-2 family induced downstream of MAPK signaling [40]. These data show that SFN-induced toxicity and MAPK signal inhibition in TNBC cells is associated with decreased cell-cycle entry and pro-survival activity.

### U0126-induced cytotoxicity correlated with MAPK signal inhibition in TNBC cells

Given our results with SFN, we predicted that targeting Mek, a key downstream substrate of Raf, would produce comparable cytotoxic effects and correspondingly similar levels of MAPK signal inhibition. Surprisingly, the allosteric Mek-inhibitor U0126 was dramatically less cytotoxic than SFN, achieving only 60% *K_Eff_* at concentrations as high as 100 μM (Figure 2D). Paradoxically, this poor cytotoxicity did not correlate with weak MAPK signal inhibition, but with a strong pErk inhibition profile similar to that produced by SFN. Intriguingly, Mek phosphorylation increased at early time points after U0126 challenge, and then returned to baseline levels; with the exception of a transient suppression at the 8 hour time point (Figure 2E). These observations indicate dynamic effects arising from Erk-mediated feedback may be occurring upstream of Mek [41–43]. Importantly, these feedback effects correlated with poor U0126 cytotoxicity, but not with Erk phosphorylation, which was effectively suppressed by Mek inhibition.

### U0126-induced cytotoxicity correlated with suppressive effects on CyclinD1, Rb and Mcl1 signaling

Expression of Cyclin D1 was relatively unaffected by U0126, and remained relatively stable throughout the 24 hours of observation (Figure 2F). In contrast, Rb phosphorylation was suppressed after 1 hour. Interestingly, Cyclin D1 and pRb both showed slight recovery at 8 hours, which correlated with the lowest levels of phosphorylated Mek (Figure 2E). Mcl-1 inhibition by U0126 was generally similar in magnitude to that achieved by SFN, but differed slightly in the pattern of suppression. While SFN-induced Mcl-1 inhibition became apparent at 4hr and beyond, U0126-induced Mcl-1 suppression was seen throughout the 24 h treatment period, but showed a transient recovery at 2 hours (Figure 2F).

ERL- SFN- and U0126-induced MAPK inhibition was also assessed at the single cell level by immunofluorescence microscopy analysis of phosphorylated Erk. Consistent with biochemical analysis, ERL (EGFR) failed to suppress pErk levels, while U0126 (Mek) and SFN (Raf) inhibition resulted in readily apparent reductions of pErk staining (Supplementary Figure 1).

### U0126 and SFN additively enhanced LPN-induced cytotoxicity in TNBC cells

**Figure 3:**
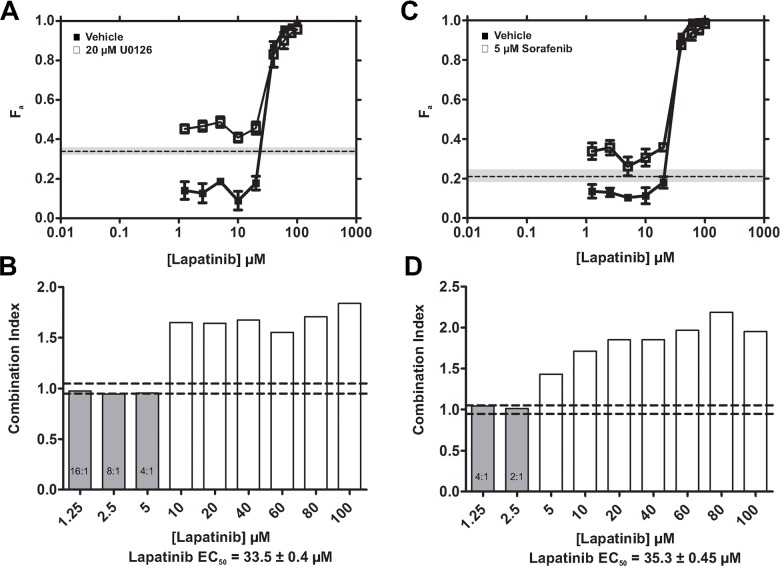
LPN-U0126 and LPN-SFN combinations exhibit additive cytotoxicity **(A, C)** The fraction of cells killed (F_a_ ± SEM) by increasing concentrations of LPN in the presence of DMSO (vehicle) or a fixed concentration of U0126 (20 μM) (A), or SFN (5 μM) (C). The fraction of cells killed by U0126 or SFN alone at these fixed concentrations is shown for comparison [dashed lines; grey shading (± SEM)]. **(B, D)** MDE-CI analysis of drug interactions with LPN + U0126 (B) or LPN + SFN. Shown are combination indices (CI) as a function of LPN concentrations. Grey and white bars denote additive (CI = 0.9-1.1) or antagonistic interactions (CI > 1.1), respectively. No synergistic interactions (CI < 0.9) were observed. Additive ratios (U0126 + LPN or SFN + LPN) are shown within grey bars, and the LPN *EC*_50_ values are indicated. Data are representative of 3 independent experiments.

We next tested combined targeting of EGFR/HER2 and downstream components of the Ras-MAPK pathway by using LPN-U0126 and LPN-SFN combinations. Fixed doses of U0126 (Figure 3A) or SFN (Figure 3C) were added to varying concentrations of LPN. MDE-CI analyses (see Materials and Methods) indicated that LPN-U0126 (Figure 3B) and LPN-SFN (Figure 3D) combinations exhibited additive cytotoxicity at low LPN concentrations; with U0126 potentiating the efficacy of LPN at ratios ranging from 16:1 to 4:1; while SFN potentiated LPN at ratios ranging from 4:1 to 2:1.

### U0126 synergized with SFN to enhance cytotoxicity in TNBC cells

**Figure 4:**
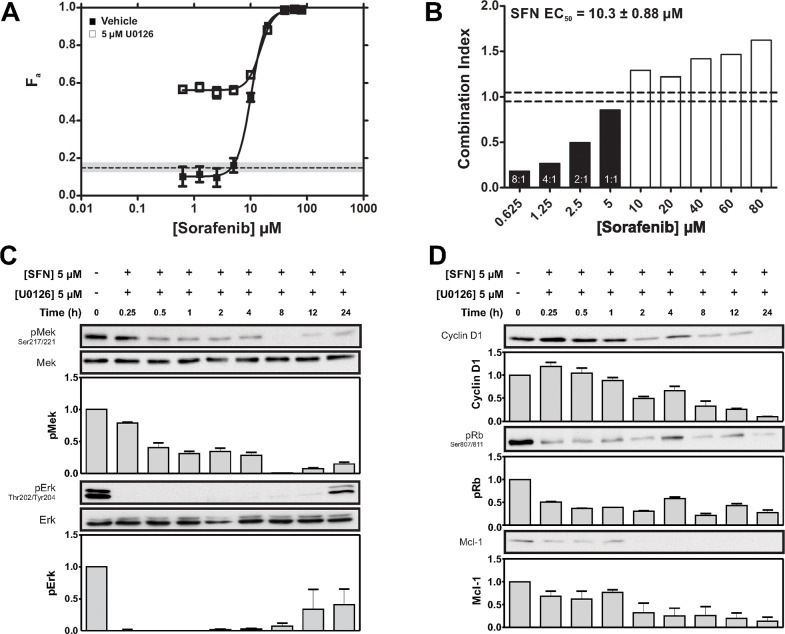
Synergistic potentiation of SFN-induced cytotoxicity by U0126 **(A)** Fraction of MDA-MB-231 cells killed (F_a_ ± SEM) by increasing concentrations of SFN in the presence of DMSO (vehicle) or at a fixed U0126 concentration (5 μM; dashed lines; grey shading ± SEM). **(B)** MDE-CI analysis of drug interactions in the panel A. Shown are combination indices (CI) as a function of SFN concentration. Black and white bars denote synergistic (CI < 0.9) or antagonistic interactions (CI > 1.1), respectively. Synergistic ratios (U0126 + SFN) and SFN *EC_50_* values are indicated. Data are representative of 3 independent experiments. **(C)** MDA-MB-231 cells were treated with SFN (5 μM) and U0126 (5 μM) for the indicated times and pMek and pErk were assessed by IB. Representative IBs and density analysis of pMek and pErk IBs are shown. pErk and pMek are expressed as a ratio of Mek and Erk intensity values, respectively (mean ± SEM; pMek/Mek, *n* = 2; pErk/Erk, *n* = 2). **(D)** MDA-MB-231 cells were treated with SFN (5 μM) and U0126 (5 μM) for the indicated times. Representative IBs showing Rb signaling (as assessed by S807/S811 phosphorylation as well as Cyclin D1 and, Mcl-1 expression are shown. Corresponding density analyses of Rb, Cyclin D1 and Mcl-1 IBs expressed as a ratio of RasGAP intensity levels are shown below (mean ± SEM; pRb/RasGAP, *n* = 2; Cyclin D1/RasGAP *n* = 2; Mcl-1/RasGAP, *n* = 2).

We next tested the effects of co-targeting nodes within the Ras-MAPK pathway. Increasing concentrations of SFN were combined with a fixed dose of 5 μM U0126; a concentration of U0126 that induced less than 20% cell killing when used alone. Surprisingly, marked enhancement in cytotoxic efficacy was apparent at SFN concentrations below its *EC*_50_ (< 10 μM) (Figure 4A). This enhancement significantly exceeded the summed effects of the individual drugs. MDE-CI analysis indicated synergy between U0126 and SFN at ratios ranging from 8:1 to 1:1 (Figure 4B). Synergy was most pronounced at the highest U0126:SFN ratios; thus 0.625 μM SFN (which induced 10% cell death in isolation), was potentiated 6-fold by the addition of 5 uM U0126 (which induced 20% cell death in isolation). These data also show that higher degrees of synergy occur at specific combination ratios.

### Combined inhibition of Raf and Mek maximally inhibited MAPK signaling

We next assessed the effect of SFN-U0126 combinations on the levels of pErk, pMek, Cyclin D1, pRb and Mcl-1. Cells growing in standard media were treated with 5 μM U0126 and 5 μM SFN (ratio 1:1) for up to 24 hours. This combination produced more robust inhibition of pErk than either drug alone (compare Figure 4C with Figures 2B, 2E). Surprisingly, in the case of pMek, the combination produced effects that were intermediate between those observed with either SFN or U0126 alone. Cyclin D1 displayed improved inhibition by the combination, compared to the effects of either drug alone (compare Figure 4D with Figures 2C, 2F). Rb phosphorylation and expression of the pro-survival factor Mcl-1 were also inhibited by the combination to a greater degree than with either drug alone (compare Figure 4D with Figures 2C, 2F).

### Therapeutic index assessment of Raf and Mek inhibition in TNBC cells

**Figure 5:**
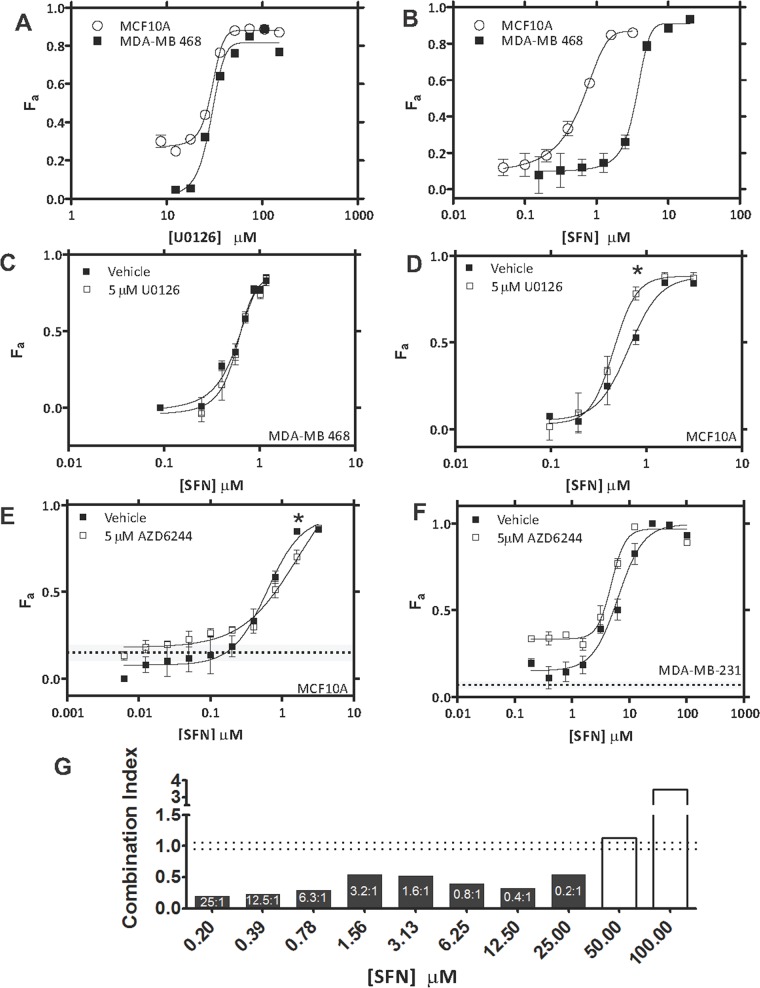
Assessment of therapeutic index for U0126/AZD6244 – SFN combinations **(A)** Cells were treated with varying concentrations of U0126 and viability was assayed after 72 h. U0126 *EC*_50_ was estimated to be 28.7 ± 1.0 μM in MDA-MB-468 cells and 27.6 ± 1.1 μM in MCF10A cells. Cytotoxicity profiles are representative of 3 independent experiments. **(B)** Cells were treated with varying concentrations of SFN and viability was assayed after 72 h. SFN *EC*_50_ was estimated to be 3.6 ± 0.3 μM in MDA-MB-468 cells and 0.5 ± 0.2 μM in MCF10A cells. Cytotoxicity profiles are representative of 3 independent experiments. **(C)** Fraction of MDA-MB-468 cells killed (F_a_ ± SEM) by increasing concentrations of SFN in the presence of DMSO (vehicle) or at a fixed concentration of U0126 (5 μM). **(D)** Fraction of MCF10A cells killed (F_a_ ± SEM) by increasing concentrations of SFN in the presence of DMSO (vehicle) or at a fixed concentration of U0126 (5 μM). **(E)** Fraction of MCF10A cells killed (F_a_ ± SEM) by increasing concentrations of SFN in the presence of DMSO (vehicle) or at a fixed concentration of AZD6244 (5 μM; dashed lines; grey shading (± SEM)). **(F)** Fraction of MDA-MB-231 cells killed (F_a_ ± SEM) by increasing concentrations of SFN in the presence of DMSO (vehicle) or at a fixed concentration of AZD6244 (5 μM; dashed lines; grey shading (± SEM)). **(G)** MDE-CI analysis of drug interactions in panel F. Shown are combination indices (CI) as a function of SFN concentration. Black and white bars denote synergistic (CI < 0.9) or antagonistic interactions (CI > 1.1), respectively. Synergistic ratios (AZD6244 + SFN) are indicated. Data are representative of 3 independent experiments.

As a surrogate for therapeutic index assessment of Raf and Mek inhibition, we compared *in vitro* cytotoxicity in TNBC cells with that in the non-transformed mammary epithelial cell line MCF10A. We also extended the analysis to a second TNBC cell line, MDA-MB-468, which overexpresses EGFR [44]. When used alone, U0126 exhibited similar cytotoxic potency in MDA-MB-468 and MCF10A cells (EC_50_ = 28.7 ± 1.0 μM and 27.6 ± 1.1 μM, respectively) (Figure 5A). Interestingly, U0126 was significantly more potent in these two cell lines than in MDA-MB-231 cells (only 60% *K_Eff_* at 100 μM, Figure 2D). Thus, cytotoxicity induced by Mek inhibition in MCF10A cells is comparable to that in MDA-MB-468, but greater than that in MDA-MB-231 cells. SFN treatment alone (Figure 5B) displayed increased cytotoxic potency in MCF10A cells (EC_50_ = 0.5 ± 0.2 μM) compared to either MDA-MB-468 (EC_50_ = 3.6 ± 0.3μM) or MDA-MB-231 cells (EC_50_ = 10.3 ± 0.9 μM; Figure 2A). These results show that individually inhibiting Mek (U0126) or Raf (SFN) failed to achieve significant therapeutic indices in these two TNBC cell lines relative to MCF10A cells.

We next evaluated the therapeutic index achievable in TNBC cells by combined inhibition of Mek and Raf. Our earlier results showed that U0126, when added at a constant concentration of 5 μM, synergized strongly with SFN at low concentration ranges in MDA-MB-231 TNBCs (0.625-5μM; Figure 4A, 4B). Remarkably, no synergy was observed under identical U0126-SFN dosing conditions in either MDA-MB-468 or MCF10A cells (Figures 5C and 5D, respectively). These data illustrate that significant therapeutic index can be achieved in MDA-MB-231 (but not MDA-MB-468) TNBC cells when Mek and Raf are co-targeted with specific dose ratios of U0126 and SFN. Thus, Raf-Mek co-targeting may provide an improved therapeutic index for some, but not all TNBC populations; which underscores the molecular heterogeneity of TNBC [1, 31]. Notably, MDA-MB-231 cells harbour activating KRAS and BRAF mutations, while MDA-MB-468 cells over-express EGFR [45].

### AZD6244 synergized with SFN to enhance cytotoxicity in MDA-MB-231 cells

U0126 is an early generation high-specificity allosteric Mek1/2 inhibitor. The more recent generation Mek inhibitor Selumitinib (AZD6244; ARRY-142886), is a selective non-ATP competitive inhibitor of Mek1/2 which has demonstrated anti-tumorigenic properties in preclinical studies of colorectal carcinoma, non-small cell lung cancer (NSCLC) melanoma and BC [11, 23, 24]. AZD6244 has recently entered clinical trials (NCT00890825) where it improved overall survival (OS) and PFS in advanced *KRAS*-mutant NSCLC patients [25]. Given the established application of AZD6244 in the clinic, we next wanted to test Raf-Mek co-targeting using AZD6244-SFN combinations. We focused on MDA-MB-231 cells in which U0126-SFN combinations exhibited a positive therapeutic index when compared to MCF10A cells (see above). Adding 5 μM AZD6244 to varying combinations of SFN produced cytotoxicity profiles in MCF10A cells that were similar to those using SFN alone (Figure 5E). In contrast, the cytotoxicity profiles produced in MDA-MB-231 cells were significantly different (Figure 5F). As observed with U0126-SFN combinations (Figure 4A), AZD6244-SFN combinations exhibited marked enhancement in cytotoxicity at SFN concentrations below its *EC*_50_ (6.3 ± 1.2 μM) (compare Figure 5F with Figure 4A). Unlike the U0126-SFN combination, greater cytotoxicity was also achieved at concentrations that approached saturation, and that well exceeded the SFN EC_50_ (6.3 ± 1.2 μM). These cytotoxic enhancements were apparent as an upward and leftward shift in the AZD6244-SFN combination curve (Figure 5F), whereas the combination curve was limited to an upward – though more pronounced – shift for the U0126-SFN combination profile (Figure 4A). MDE-CI analysis concurred with the broad effect of AZD6244-mediated enhancement in cytotoxicity across most of the SFN concentration spectrum (Figure 5G). Synergy was indicated between AZD6244 and SFN at ratios ranging from 25:1 – 0.2-1, which far exceeded the range observed with U0126-SFN combination (8:1 to 1:1; Figure 4B). Expectedly, these data confirm that, like U0126-SFN combinations, AZD6244-SFN combinations also display synergy; and interestingly, while the synergy is reduced in magnitude, its effect extends across a wider SFN concentration range.

### SFN-AZD6244 in combination maximally suppresses tumor growth *in vivo*

**Figure 6:**
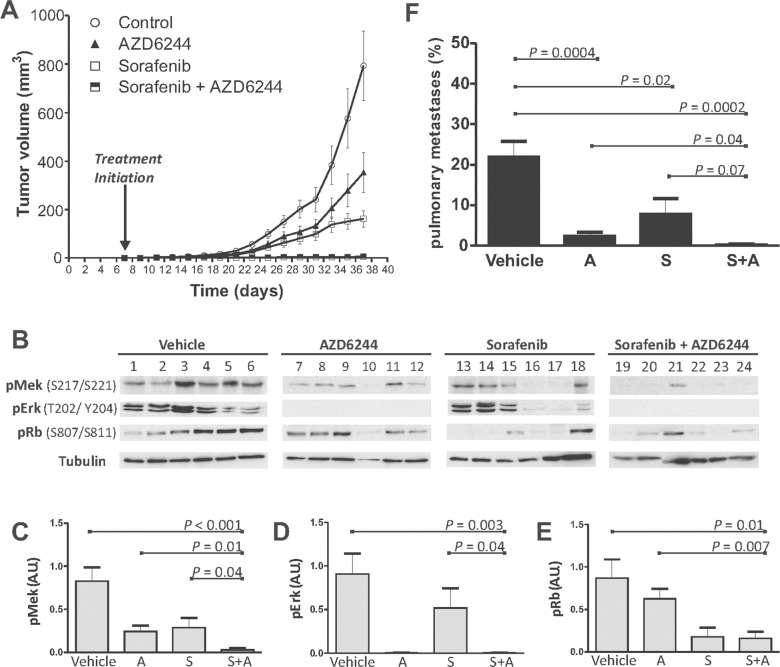
Combined treatment with SFN and AZD6244 results in enhanced tumor suppression *in vivo* **(A)** Xenograft tumor growth profiles of vehicle (control) or drug-treated mice (6 mice/cohort). Shown are caliper measurement-based estimates of mean Tumor Volume ± SEM. Control versus SFN, AZD6244 or SFN + AZD6244 (*p*<0.0001; 2-way ANOVA). SFN + AZD6244 versus SFN or AZD6244 (*p*<0.0001; 2-way ANOVA). **(B)** IB analysis of tumors assessing phosphorylation of Mek, Erk and Rb in response to Vehicle, AZD6244, SFN or SFN plus AZD6244. Tubulin IBs were used to assess loading. **(C**-**E)** Densitometry analyses of the average intensity of phosphorylated Mek (C), Erk (D) and Rb (E). Shown is the mean ± SEM intensity expressed as a ratio of tubulin intensity. Significant *P* values (t-TEST) are indicated. Arbitrary Units (A.U.) **(F)** Pulmonary metastatic tumor burden was assessed by morphometric measurements using ePATHOLOGY IMAGESCOPE and IMAGEPRO software. Quantification of the ratio (± SEM) of infiltrative metastatic tumor clusters to normal lung parenchyma was performed (*n*=6 for each cohort). Significant *P* values (t-TEST) are indicated. For images of individual tumors and sections, see Supplementary Figure 2, 3.

We next explored combined Raf and Mek inhibition using SFN and AZD6244 in a mouse orthotopic xenograft model. MDA-MB-231 cells were engrafted into mammary glands of BALB/c *RAG2^−/−^|IL2Rγc^−/−^* mice and SFN or AZD6244 alone, or in combination was administered. Each drug significantly reduced the rate of tumor development relative to control mice (Figure 6A); and the SFN-AZD6244 combination almost eliminated tumor growth (Figure 6A, Supplementary Figure 2). No significant adverse reactions were observed, suggesting that combined Raf and Mek inhibition by SFN and AZD6244 at concentrations sufficient to achieve inhibition of tumor growth *in vivo* may be well tolerated.

### SFN-AZD6244 combinations maximally inhibited phosphorylation of Mek, Erk and Rb *in vivo*

Molecular mechanisms underlying tumor suppression were further explored by measuring phosphorylation of Mek, Erk and Rb in resected tumors (Figure 6B). Mek phosphorylation in tumor samples was reduced by SFN alone (p=0.021) or AZD alone (p=0.007), and to the greatest extent with the combination of both drugs (p<0.001) (Figure 6C). In comparison to those treated with individual drugs, the SFN-AZD6244 combination showed greater inhibition of Raf-mediated Mek activation, as assessed by *in vivo* phosphorylation of Mek at S217/S221 (Figure 6C). Erk phosphorylation *in vivo* was essentially abolished with either AZD6244 alone, or the AZD6244-SFN combination (Figure 6B, 6D); whereas SFN alone reduced Erk phosphorylation in only 2 of 6 tumors (Figure 6B), and no significant difference was apparent across the whole cohort (Figure 6D). This refractory behaviour of Erk to *in vivo* SFN challenge is consistent with our previous observations [46]. In contrast to Erk, Rb phosphorylation was not affected by AZD6244, but was inhibited by SFN (p=0.017) or the SFN-AZD6244 combination (p=0.01) (Figure 6E). The SFN-AZD6244 combination did not achieve greater inhibition of Rb phosphorylation relative to SFN alone, but was more effective than AZD6244 alone (Figure 6E).

### Reduction in metastatic pulmonary tumor burden by SFN and AZD6244

Lungs resected at the end of treatment were examined for histological evidence of metastases (Supplementary Figure 3). Semi-quantitative assessment of lung sections using ePATHOLOGY IMAGESCOPE software (see Materials and Methods) verified that the metastatic load in the lungs was significantly reduced by either SFN (p=0.02) or AZD6244 (p=0.0004) alone, but the SFN-AZD6244 combination achieved the greatest effect on pulmonary metastases (p=0.0002) (Figure 6F; Supplementary Figure 3).

## DISCUSSION

Hyperactive Ras-MAPK signaling is frequently observed in cancer [11]. Oncogenic drivers of this pathway include RTKs such as EGFR and HER2, as well as downstream nodes including Ras, Raf and Mek. Mutations or amplified expression in lung cancer, melanoma and HER2^+^ BC provides rationale for targeting these nodes. While comparable genetic evidence is not typical in TNBC, frequently observed hyperactive Ras-MAPK signaling argues that targeting nodes in this pathway could provide therapeutic benefit [30].

Here, we tested the cytotoxic effects of targeting EGFR/HER2 receptors, as well as downstream Raf and Mek signaling proteins in the MDA-MB-231 model of TNBC. The weak cytotoxic effect of the EGFR inhibitor ERL correlated with unperturbed MAPK signaling activity (Figure 1A, 1B). More cytotoxicity was achieved by targeting both EGFR and HER2 with LPN (Figure 1C), and this correlated with inhibition of Mek and Erk phosphorylation (Figure 1D). These data suggest that HER2, likely in the context of EGFR/HER2 heterodimers, is a major route of RTK signaling to the Ras-MAPK pathway in MDA-MB-231 cells. Since these cells harbour an activating *KRAS* mutation [47], incomplete suppression of Ras-MAPK signaling by LPN-mediated inhibition of the upstream RTKs was expected.

In line with studies showing anti-tumorigenic effects of the Raf inhibitor SFN on tumors with activated Ras-MAPK signaling [48], we found this drug to be highly cytotoxic to MDA-MB-231 cells. Indeed, SFN exhibited a greater *in vitro* potency relative to LPN (*EC*_50_ of 10.3 μM vs 38.8 μM, respectively; Figures 2A vs 1C); and this also correlated with a greater inhibition of Mek and Erk phosphorylation relative to that of LPN (Figures 2B vs 1D). This is consistent with reports that the Ras-MAPK pathway plays a critical role in cell survival [49]. Interestingly, SFN also suppressed the phosphorylation of Rb, suggesting an inhibitory effect on CDK activity. The expression of Mcl-1, a pro-survival member of the Bcl-2 family, was also inhibited, which could also contribute to the mechanism of SFN in promoting apoptosis [50, 51].

Inhibition of Ras-MAPK signaling using the Mek inhibitor U0126 failed to achieve maximal cytotoxicity despite marked suppression of Erk phosphorylation (Figure 2D, 2E). Of note, while Mek inhibition with U0126 resulted in the expected reduction in phosphorylation of its substrate Erk, it was also associated with increased Mek phosphorylation (Figure 2E). This observation is consistent with loss of Erk-mediated negative feedback [52–56], which would be expected to result in increased upstream signaling activity, including Raf-mediated phosphorylation of Mek. Loss of Erk-mediated negative feedback may be a key underlying factor in the observed cytotoxic insensitivity to Mek inhibition (Figure 2D). Moreover, loss of negative feedback may promote Raf-mediated Mek/Erk-independent signaling mechanisms that may contribute to survival. Activation of the parallel PI3K pathway is one such mechanism which has been described in basal BC [49]. Another mechanism involves direct Raf interactions with Rb, resulting in hyper-activation of the Rb-E2F pathway [57]. Upstream Raf activation through loss of Erk negative feedback could also lead to CDC25A activation, and CDK activation [58]. Consistent with these possibilities, combined Raf and Mek inhibition achieved greater cytotoxicity than targeting either kinase alone. MDE-CI analyses indicated that U0126 synergistically enhanced the cytotoxic effects of SFN (Figure 4B). Potentiation by U0126 was most pronounced at sub-*EC*_50_ SFN concentrations (< 10 μM) at ratios ranging from 8:1 to 1:1, showing added benefits of drug combination to reduce doses, while achieving optimal therapeutic efficacy. We speculate that compensatory inhibition of orthogonal Mek-independent c-Raf survival pathways by SFN may constitute the basis of the synergy observed with U0126. In contrast, LPN-U0126 and LPN-SFN combinations only yielded additive cytotoxicity effects (Figure 3). SFN-U0126 combinations were more effective at inhibiting the phosphorylation of Erk and Rb, as well as reducing Cyclin D1 expression, than either drug alone (Figures 4C, 4D vs 2B-2F). Combined Raf and Mek inhibition with SFN and U0126 also led to reduced Mcl-1 expression. SFN cytotoxicity has been associated with down-regulation of Mcl-1 in epithelial and leukemic cancer cell lines [50, 51]. The mechanism by which SFN suppresses Mcl-1 expression remains unclear, although it may be independent of MAPK signaling [51].

Enhanced tumor growth suppression by combined SFN and AZD6244 was associated with reductions in Mek, Erk and Rb phosphorylation (Figure 6B–6E). AZD6244 was highly effective at blocking Erk phosphorylation in tumors, suggesting robust *in vivo* inhibition of Mek (Figure 6B, 6D). Interestingly, Mek phosphorylation in tumors was also significantly suppressed by AZD6244 alone (Figure 6B, 6C), suggesting that increased Raf-mediated Mek phosphorylation through loss of Erk-mediated negative feedback inhibition may be more difficult to detect *in vivo* than it is *in vitro* (Figure 2E).

*In vivo* Rb phosphorylation levels were unaffected by AZD6244 alone, despite Erk inhibition (Figure 6B, 6E). We speculate that this could be due to Erk-independent Rb activation through Raf [57]. Consistent with this idea, marked reduction of Rb phosphorylation was noted in SFN and SFN-AZD6244 treated tumors (Figure 6B, 6E). Interestingly, Erk phosphorylation *in vivo* was not significantly suppressed by SFN alone. This has been previously reported in liver, colon, and breast tumor models, indicating *in vivo* anti-tumorigenic effects of SFN may be Erk-independent [33, 46, 59–61].

Marked reduction in pulmonary metastatic burden was noted in SFN, AZD6244, and SFN-AZD4244 treated groups (Figure 6F, Supplementary Figure 3). Consistent with our previous study combining SFN and flavopiridol [46], SFN alone significantly suppressed pulmonary metastatic burden. The anti-tumorigenic effects of AZD6244 on tumor growth at the orthotopic site correlated with significantly reduced pulmonary metastases (Figure 6A, 6F, Supplementary Figures 2, 3).

In a phase I trial of AZD6244 with advanced cancer patients, inhibition of Erk phosphorylation in peripheral blood mononuclear cells was associated with anti-tumorigenic effects [62]. This correlates with our *in vivo* findings of significant suppression of Erk phosphorylation in AZD6244-treated tumors. In addition to MAPK signaling inhibition, an increase in cleaved PARP positive cells has been described in AZD6244 treated tumor models, showing that AZD6244 induces apoptosis [60]. Ki-67 immunohistochemistry analyses on tumors showed that SFN also significantly reduced mitotic cells in comparison to AZD6244, confirming that SFN reduces cell proliferation [60]. The anti-proliferative effects of SFN could be attributed to greater tumor growth suppression in comparison to AZD6244 treatment alone, although no significant difference was noticed in the reduction of pulmonary metastases between SFN and AZD6244 treated groups (Figure 6F, Supplementary Figure 3).

A phase II trial (SOLTI-0701) has shown that treatment of advanced HER2 negative BC patients with SFN resulted in prolonged PFS [21]. Another promising finding from a phase II trial of HER2 negative patients with disease progression during or after treatment with the angiogenic inhibitor Bevacizumab (NCT00493636) showed that treatment with SFN resulted in increased PFS [22].

TNBC is the most aggressive subtype of BC. Surgery and adjuvant or neoadjuvant chemotherapy are currently used to treat these patients. Although gene amplification and activating mutations in genes encoding components of the Ras-MAPK pathway (including *HRAS, BRAF, MEK1 and MEK2*) are rarely seen in TNBC, the molecular signature of these tumors frequently indicates activation of this pathway [63]. Due to high frequency of EGFR expression in TNBC, Ras-MAPK signaling is often upregulated [1, 35]. However, there are no currently approved targeted agents designed to inhibit this pathway in TNBC [30]. These observations provide strong rationale for exploring the use of inhibitors of key components of the Ras-MAPK pathway in TNBC. Mek inhibition has shown promising results in preclinical models of TNBC; however, decreased Erk activity due to Mek inhibition has sometimes been associated with increased PI3K/Akt signaling [64]. AKT activity was very low in the MDA-MB-231 model system used in this study, and no increased AKT phosphorylation was observed in tumors from drug treated mice (data not shown). This does not preclude the potential for in-parallel inhibition of the Ras-MAPK and PI3K-Akt-mTOR pathways in TNBC; and indeed, there are several ongoing phase I and II trials investigating this combination in solid tumors (reviewed in [65]); but to our knowledge, none of these trials are in TNBC.

MDA-MB-231 cells have both *KRAS* and *BRAF* mutations, which are observed in 1-2% of primary TNBC samples [66]. Activating mutations of this type are associated with cellular transformation [67]; which is consistent with our expectation that they contribute to the tumorigenic and metastatic behaviour of the MDA-MB-231 model. This cell line has been widely used to explore genes and signaling pathways that contribute to tumorigenesis [68–72]; including TNBC, where activation of the Ras-MAPK pathway is frequently observed [30]. However, TNBC is genetically heterogeneous, and MDA-MB-231 should not be considered as a generally representative model for this breast cancer subtype [73].

In summary, this study highlights the potential of targeting multiple in-series nodes of the Ras-MAPK pathway to improve treatment in TNBC and suggests that combined inhibition of Erk-dependent and Erk-independent signaling (using Mek and Raf inhibitors, respectively), may be achievable with currently available drugs.

## MATERIALS AND METHODS

### Cell lines and reagents

MDA-MB-231 cells were provided by Dr. Peter Siegel (McGill University, Montreal, QC) and cultured in Dulbeccos modified essential medium supplemented with 10% fetal bovine serum (Gibco Life Technologies, Burlington, ON). SFN, LPN, and erlotinib were obtained from Toronto Research Chemicals (Toronto, ON). U0126 and PD98059 were from Sigma-Aldrich (Oakville, ON). AZD6244 (selumitinib) was from Selleckchem (Houston, TX). Antibodies were from Cell Signaling Technology (Beverly, MA).

### Cytotoxicity assay

Two × 10^4^ cells per well in 96-well plates were treated with varying concentrations of erlotinib, LPN, SFN or AZD6244 for 72 h under standard culture conditions. For drug-combinations, cells were treated with increasing concentrations of a primary drug in the presence of a fixed concentration of a secondary drug. Cell viability was assessed by metabolic activity using 3-(4,5-dimethylthiazol-2-yl)-2,5-diphenyltetrazolium bromide (MTT assay; Sigma-Aldrich). Drug interactions were analyzed using multiple drug-effect/combination index (MDE-CI) isobologram analysis (CALCUSYN software; [74]); where CI values < 0.9, 0.9-1.1 and > 1.1 indicate, synergy, additivity and antagonism, respectively. F_a_ represents the proportion of cells killed or no longer viable as a result of drug treatment; calculated as F_a_ = *A*_570_control – *A*_570_ treated / *A*_570_ control [15].

### Signaling experiments

One x 10^7^ cells per 10cm plate were treated with erlotinib, LPN, SFN and U0126 alone or in combination at the indicated times and concentrations in standard culture media. After treatment, cells were lysed in radioimmunoprecipitation assay (RIPA) buffer, supplemented with protease inhibitorsand clarified by centrifugation (12.000 *g*, 10 min). Equal quantities of protein were separated by sodium-dodecyl-sulfate-polyacrylamide gel electrophoresis and assayed by immunoblotting (IB) using the indicated antibodies. For immunofluorescence studies, cells were seeded on 12mm glass coverslips (Fisher Scientific, ON), fixed in 3% paraformaldehyde, permeabilized with 0.2% TritonX-100 (Fisher Scientific, ON), and blocked in 3% bovine serum albumin. Cells were subsequently stained with the indicated primary antibody and appropriate AlexaFluor 546 secondary antibody. Alexa 488 Phalloidin and 4′,6-diamidino-2-phenylindole (DAPI) were used to stain F-actin and nuclei, respectively. Cell images were captured on a Leica TCS-SP2 inverted confocal microscope (Leica Microsystems, ON).

### Orthotopic xenograft model of TNBC

MDA-MB-231 cells growing in logarithmic phase were resuspended in 1:1 phosphate buffered saline (PBS):Matrigel (Sigma-Aldrich) solution. Two x 10^6^ cells were injected into the right inguinal mammary gland of BalbC *RAG2^−/−^|IL2Rγc^−/−^* mice (kindly provided by Dr. M. Ito, Central Institute of Experimental Animals, Kawasaki, Japan). SFN was dissolved in H_2_O containing 12.5% Cremophor EL (Sigma-Aldrich) and 12.5% ethanol and administered by oral gavage (*og*). AZD6244 was dissolved in water and administered by *og*. Mice were randomized into four cohorts (six mice per cohort). The control cohort received PBS (*og*) and treatment cohorts received either SFN (30 mg/kg, *og*), AZD6244 (20 mg/kg, *og*) or SFN-AZD6244 (30 and 20 mg/kg, respectively) [60, 61, 75]. Treatment was initiated 7 days post-engraftment and continued every 2 days for 30 days. Tumor sizes were measured every 2 days using calipers. After 37 days, and 1 h after the last drug treatment, tumors were resected, bisected and either lysed in RIPA buffer for IB analysis or formalin-fixed and paraffin-embedded (FFPE) for histological analysis. IBs were quantified using IMAGEJ software [76].

### Pathological assessment of pulmonary metastatic tumor burden

Whole lungs were resected 37 days post-engraftment and prepared in FFPE tissue blocks. Two 4 μm sections (200 μm apart) from each lung were assessed blindly by two independent clinical pathologists. Relative metastatic burden was morphometrically measured using ePATHOLOGY IMAGESCOPE (Aperio, Vista, CA) and IMAGEPRO software (MediaCybernetics, Rockville, MD) [77]. The area infiltrated by cancer cells relative to the total lung area was calculated to determine the % metastatic burden. Mice were housed in the Queen's University Animal Care Facility. All procedures were approved by the institutional animal care committee according to the guidelines of the Canadian Council on Animal Care.

## SUPPLEMENTARY FIGURES



## Abbreviations

AZD6422: selumitinib
BC: breast cancer
EGFR: epidermal growth factor receptor
ERL: erlotinib
ER: estrogen receptor
FFPE: formalin fixed and paraffin embedded
HER2: human epidermal growth factor receptor-2
IB: immunoblotting
K_eff_: killing efficiency
LPN: lapatinib
OS: overall survival
PFS: progression-free survival
PR: progesterone receptor
RTK: receptor tyrosine kinase
SFN: sorafinib
TNBC: triple negative breast cancer
