# Supplementary material for: Combined targeting of Raf and Mek synergistically inhibits tumorigenesis in triple negative breast cancer model systems
Source: Oncotarget. 2017 Aug 24;8(46):80804–19. doi: 10.18632/oncotarget.20534 (PMC5655240; doi:10.18632/oncotarget.20534)
Supplement: Supplementary file 1 [file oncotarget-08-80804-s001.pdf]

Combined targeting of Raf and Mek synergistically inhibits tumorigenesis in triple negative breast cancer model systems

SUPPLEMENTARY MATERIALS

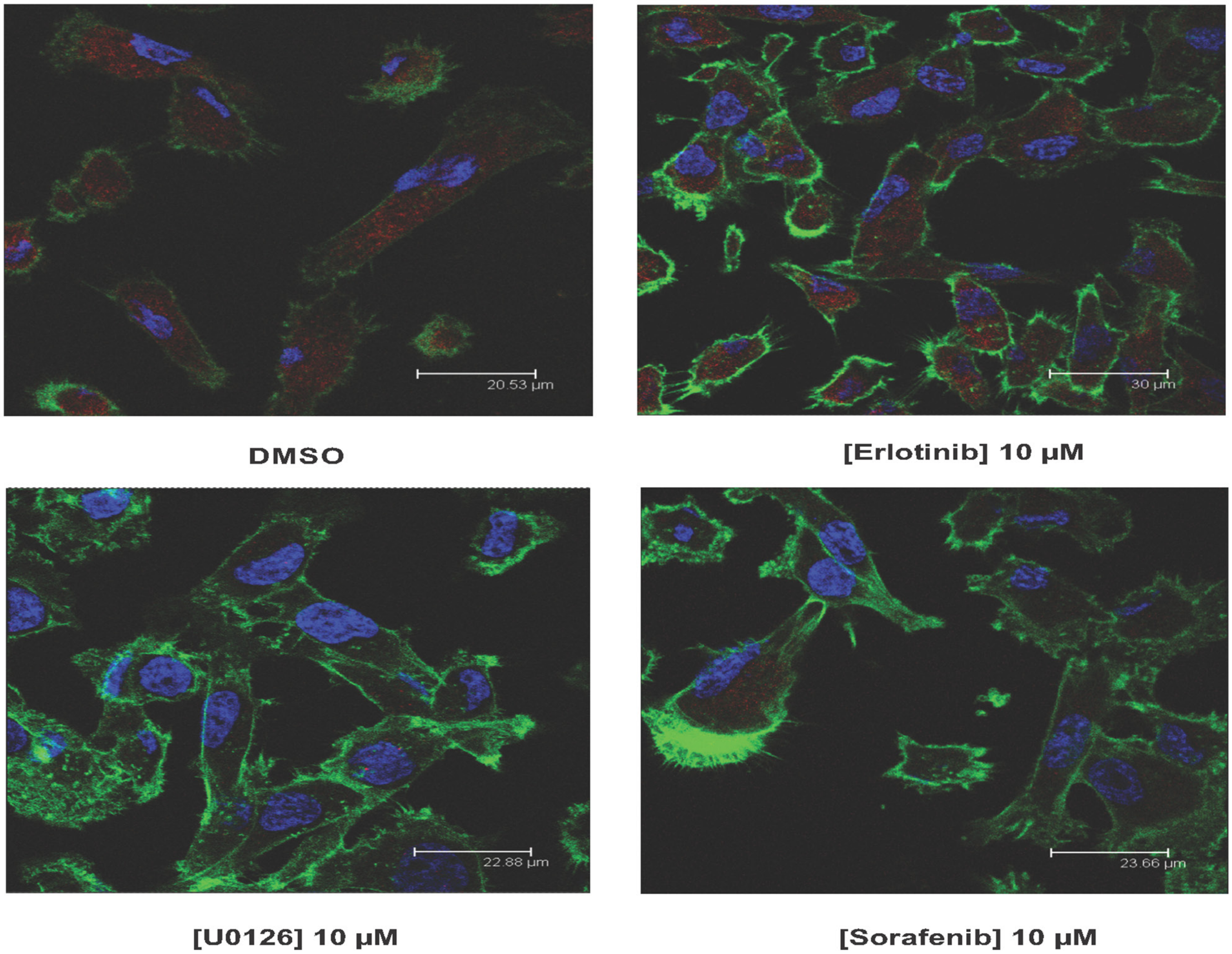

Supplementary Figure 1: Assessment of the effect of Erlotinib, SFN and U0126 on *in situ* pErk levels. MDA-MB-231 cells were treated with vehicle (DMSO), ERL, U0126, and SFN at the indicated concentrations for 1 hour. Cells were then stained for pErk (red), F-actin (green) and DAPI for nuclei (blue) and images were acquired by confocal fluorescence microscopy as described in the Materials and Methods section.

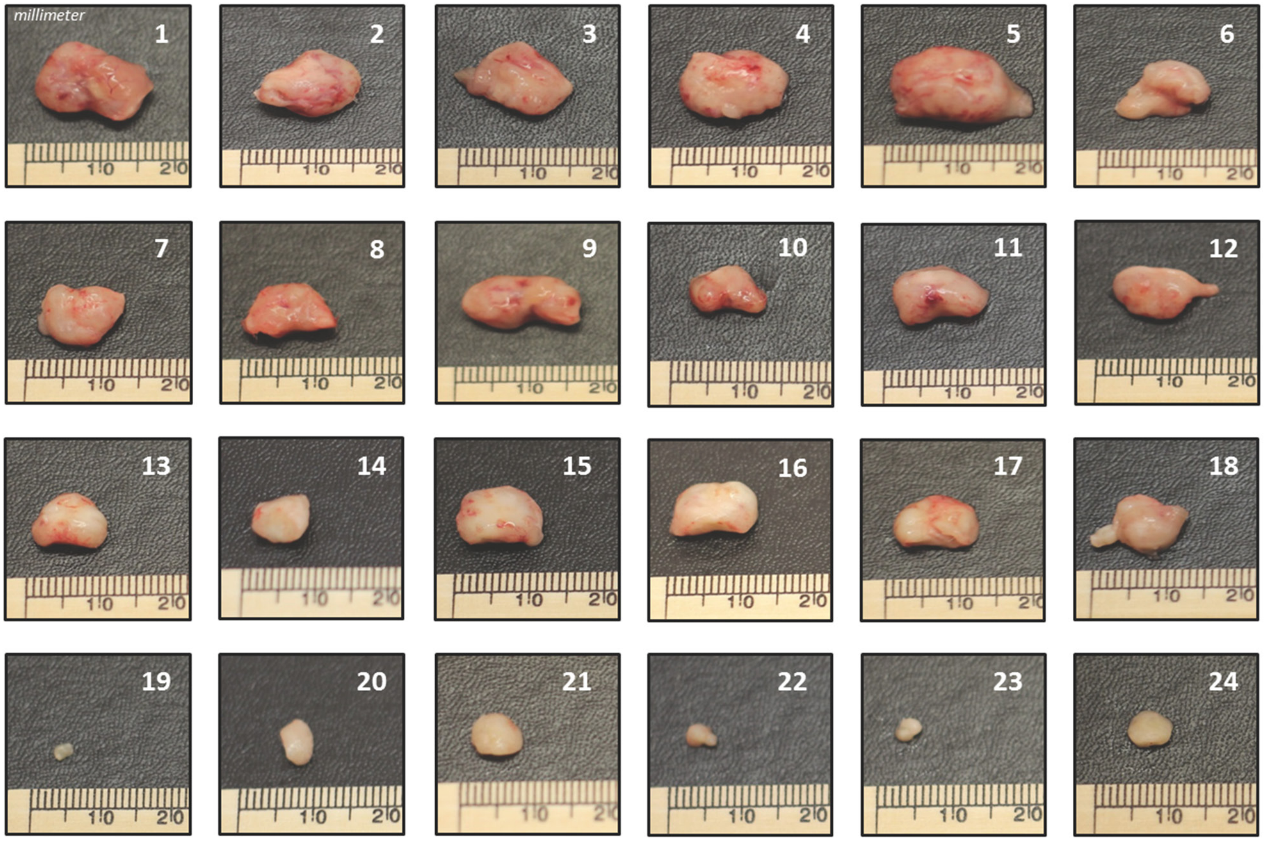

Supplementary Figure 2: Gross pathology of primary tumors resected from the orthotopic sites at day 37. (1-6) Control cohort; (7-12) AZD6244 cohort; (13-18) SFN cohort; (19-24) SFN-AZD6244 combination cohort. Unit measurement are shown in millimeters.

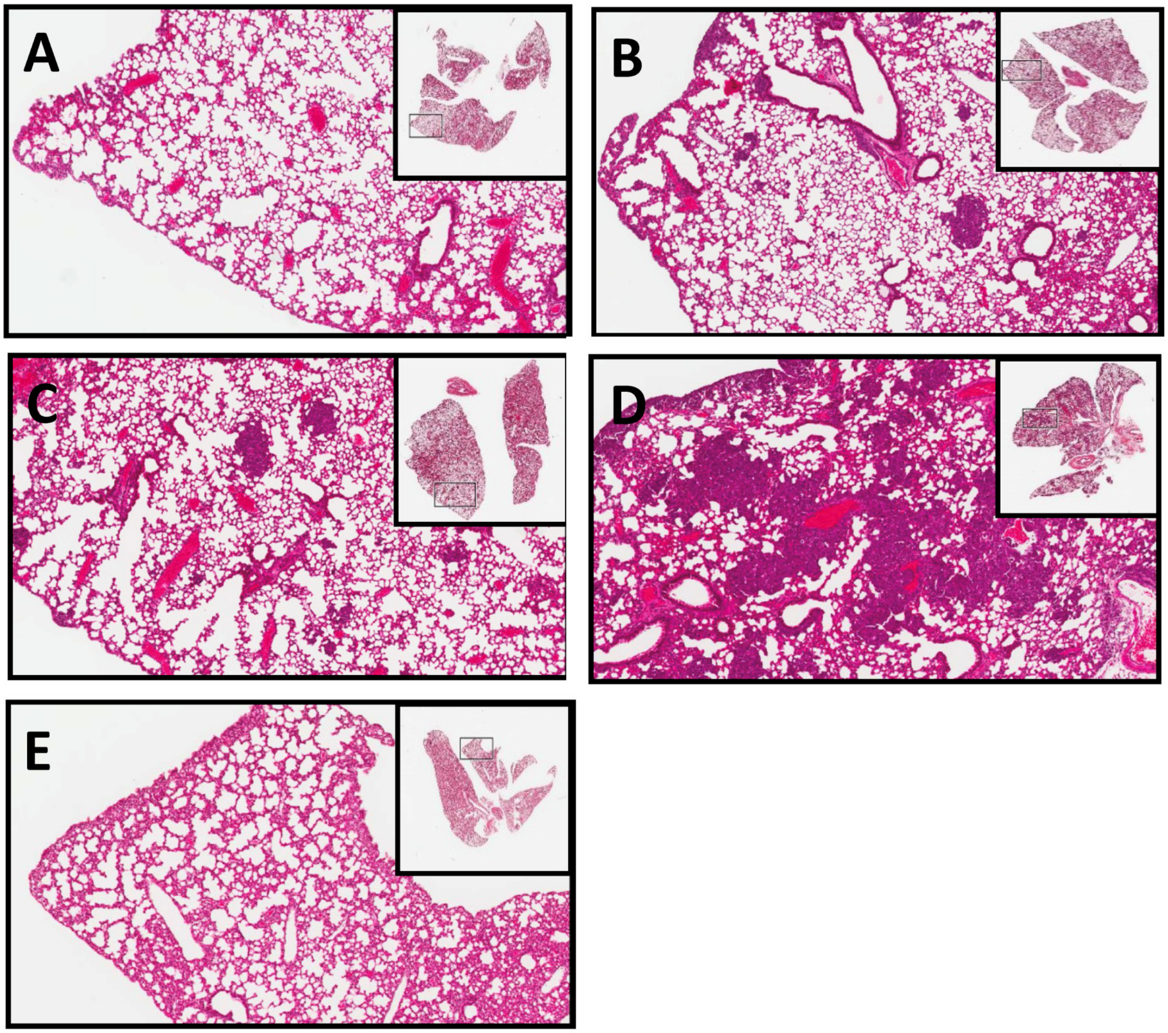

Supplementary Figure 3: Pathological assessment of pulmonary metastatic tumor load. Representative images of harvested lung sections stained with Hematoxylin and Eosin from tumor xenograft mice cohorts. Mice treated with SFN-AZD6244 **(A)** had minimal detectable metastatic tumor deposits. Mice treated with SFN **(B)** or AZD6244 **(C)** alone had a relatively low overall metastatic burden and tumors were present as isolated nests; Mice treated with the vehicle only **(D)** had diffuse involvement of the lung parenchyma with metastatic tumors arranged in coalescing nests and sheets more prominent in the subpleural and perivascular zones. Lungs from a non-xenografted and non-treated age-matched mouse is included for reference purposes **(E)**. The whole lung FFPE sections are shown in right-upper inserts with the regions corresponding to the higher power images boxed.
